# Pylorus-Preserving Versus Pylorus-Resecting Pancreaticoduodenectomy for Periampullary and Pancreatic Carcinoma: A Meta-Analysis

**DOI:** 10.1371/journal.pone.0090316

**Published:** 2014-03-06

**Authors:** Chong Yang, He-Shui Wu, Xing-Lin Chen, Chun-You Wang, Shan-Miao Gou, Jun Xiao, Zhi-Qiang He, Qi-Jun Chen, Yong-Feng Li

**Affiliations:** 1 Pancreatic Disease Institute, Union Hospital, Tongji Medical College, Huazhong University of Science and Technology, Wuhan, People's Republic of China; 2 Key Laboratory of Geriatrics of Health Ministry, Department of Geriatrics, Union Hospital, Tongji Medical College, Huazhong University of Science and Technology, Wuhan, People's Republic of China; West German Cancer Center, Germany

## Abstract

**Background:**

The aim of this meta-analysis was to compare the long-term survival, mortality, morbidity and the operation-related events in patients with periampullary and pancreatic carcinoma undergoing pylorus-preserving pancreaticoduodenectomy (PPPD) and pylorus-resecting pancreaticoduodenectomy (PRPD).

**Method:**

A systematic search of literature databases (Cochrane Library, PubMed, EMBASE and Web of Science) was performed to identify studies. Outcome measures comparing PPPD versus PRPD for periampullary and pancreatic carcinoma were long-term survival, mortality, morbidity (overall morbidity, delayed gastric emptying [DGE], pancreatic fistula, wound infection, postoperative bleeding, biliary leakage, ascites and gastroenterostomy leakage) and operation related events (hospital stays, operating time, intraoperative blood loss and red blood cell transfusions).

**Results:**

Eight randomized controlled trials (RCTs) including 622 patients were identified and included in the analysis. Among these patients, it revealed no difference in long-term survival between the PPPD and PRPD groups (HR = 0.23, p = 0.11). There was a lower rate of DGE (RR = 2.35, p = 0.04, 95% CI, 1.06–5.21) with PRPD. Mortality, overall morbidity, pancreatic fistula, wound infection, postoperative bleeding, biliary leakage, ascites and gastroenterostomy leakage were not significantly different between the groups. PPPDs were performed more quickly than PRPDs (WMD = 53.25 minutes, p = 0.01, 95% CI, 12.53–93.97); and there was less estimated intraoperative blood loss (WMD = 365.21 ml, p = 0.006, 95% CI, 102.71–627.71) and fewer red blood cell transfusions (WMD = 0.29 U, p = 0.003, 95% CI, 0.10–0.48) in patients undergoing PPPD. The hospital stays showed no significant difference.

**Conclusions:**

PPPD had advantages over PRPD in operating time, intraoperative blood loss and red blood cell transfusions, but had a significantly higher rate of DGE for periampullary and pancreatic carcinoma. PPPD and PRPD had comparable mortality and morbidity including pancreatic fistulas, wound infections, postoperative bleeding, biliary leakage, ascites and gastroenterostomy leakage. Our conclusions were limited by the available data. Further evaluations of high-quality RCTs are needed.

## Introduction

Pancreatic carcinoma is the fourth most common malignancy and is associated with an extremely poor prognosis, reflected by a median survival of <6 months and a 5-year survival of <5% [1], [2]. Currently, surgical resection provides the only hope of a cure for periampullary and pancreatic carcinoma, whereas high rates of postoperative complications remain significant causes of mortality and markedly prolonged hospitalizations [3].

Pancreaticoduodenectomy (PD) is the primary surgical treatment for patients with periampullary and pancreatic carcinoma. The pylorus-resecting PD (PRPD) operation involves removing the pancreatic head, duodenum, common bile duct, gall bladder with (or without) the distal portion of the stomach associated with the adjacent lymph nodes [4]. Pylorus-preserving PD (PPPD) is similar with the exception that the pylorus and the first portion of the duodenum are preserved and continuity is restored through a duodenojejunostomy [5]. To date, the debate continues as to whether PPPD or PRPD is better for periampullary and pancreatic carcinoma. PRPD has been advocated because peripyloric lymph nodes can be adequately dissected under direct vision and a safe surgical margin is easy to achieve during the operation, with the intent to decrease the risk of tumor recurrence and to prolong survival [6]. PPPD has been recommended because it involved a less extensive dissection and therefore is superior to PRPD in terms of perioperative blood loss and postoperative quality of life [7]. Although some clinical controlled trials have demonstrated the superiority of PPPD to PRPD, other researchers have reported that PPPD and PRPD were equally effective in terms of postoperative complications, mortality, and long-term survival [8], [9]. Furthermore, Roder JD *et al* suggested that better survival occurred after PRPD than PPPD for patients with pancreatic ductal adenocarcinoma [10]. And Lin *et al* claimed that there was no significant difference between PPPD and PRPD for the treatment of pancreatic head cancer, whereas delayed gastric empty (DGE) was more frequently encountered with PPPD than with PRPD [11]. Regarding safety, recent study reported a shorter operating time and reduced blood loss with PPPD [12], [13]. However, certain authors have argued that the PRPD technique does not increase postoperative morbidity and mortality [14].

Uncertainty remains regarding differences of survival and safety between PPPD and PRPD in cases of periampullary and pancreatic carcinoma. To further ascertain the effectiveness of the PPPD and PRPD procedures, we conducted a current, thorough literature search and meta-analysis to compare the outcomes of the two surgical procedures.

## Methods

### Data sources and searches

Two investigators (CY and XLC) searched Cochrane Library, PubMed, EMBASE and Web of Science for relevant articles published before Aug 5, 2013; no date or specific language limits were applied. NoteExpress information management software was used for a standardized record form and data collection. A sensitive search was performed using terms related to PPPD, standard Whipple's pancreaticoduodenectomy (SWPD), PRPD and randomized trials in pancreatic or periampullary cancer patients. We used the following Medical Subject Heading terms and keywords: “Whipple operation”, “pylorus”, “pancreaticoduodenectomy”, “pylorus-preserving” and “pancreatic/ periampullary tumor”. The search strategy also used text terms such as “Whipple procedure”, “standard pancreaticoduodenectomy”, “Classic duodenopancreatectomy”, “Duodenopancreatectomy”, and “Pylorus preserving pancreaticoduodenectomy” to identify relevant information. We entered Boolean operators (AND, OR, NOT) to combine or exclude search terms. The search was limited initially to publications of human randomized controlled trials (RCTs). We screened the references of the included studies and related publications. Investigators and experts in the field of pancreatic surgery were contacted to ensure that all the relevant studies were identified. The results were hand-searched for eligible trials. The results were verified and arbitrated by a third investigator (HSW).

### Study selection

The primary goal of this study was to determine the relative effects of PPPD and PRPD in patients with pancreatic or periampullary cancer. Therefore, we selected only those RCTs that directly compared patients with pancreatic or periampullary cancer who underwent a PPPD or a PRPD. Papers lacking a control group were excluded. Only RCTs reporting quantitative data for at least one of the following outcomes were selected for data extraction: long term survival, mortality, morbidity (including overall morbidity, DGE, pancreatic fistulas, wound infections, postoperative bleeding, biliary leakage, ascites, gastroenterostomy leakage) and operation related events (hospital stays, operating time, intraoperative blood loss, red blood cell transfusions). We excluded studies that were not published as an original paper, such as conference abstracts and letters to the editor.

### Data extraction and quality assessment

To avoid bias in the data selection process, two authors (CY and XLC) independently extracted data from the trials and subsequently compared the results. We extracted details on the sample size and extracted quantitative data on long term survival, postoperative mortality, morbidity and operation related events. Two authors selected the data independently, and any discrepancies between the authors were resolved by consensus. All the data were evaluated for internal consistency, and disagreements were resolved by via a discussion with a third author (HSW). All the authors have none academic or private relationship with the authors and institutions of the included articles. The quality was assessed using criteria such as adequate blinding of randomization, completeness of follow-up, and objectivity of outcome measurements as described previously [15].

### Statistical analysis

All the statistical analyses were performed using STATA 11.0 (STATA Corp, College Station, Texas) and RevMan 5 (http://ims.cochrane.org/revman/download). All the p-values were two-sided. We analyzed the dichotomous variables by calculating the risk ratio (RR) and corresponding 95%CI as the summary statistic, and we converted continuous data that was reported as the median and range to the mean and standard deviation using the method of Hozo [16]. For continuous outcomes, we pooled the data using the weighted mean difference (WMD). The long-term survival rates were generated by extracting events (Kaplan-Meier curves) and calculating logarithmic hazard ratios (±standard error) using the corresponding p value (log-rank test) [17]. If the data could not be extracted for the meta-analysis, we presented the data in a descriptive manner in the results.

For the meta-analysis, we used a fixed-effects (weighted with inverse variance) or a random-effects model based on the heterogeneity of the included studies [18]. For each meta-analysis, Cochran's *Q* statistic and *I* ^2^ statistics were first calculated to assess the heterogeneity among the proportions of the included trials. If the p -value was less than 0.10, the assumption of homogeneity was deemed invalid, and the random-effects model was utilized after exploring the causes of heterogeneity [19]. When the results of the two models were substantially different, the random-effects model was presented. Otherwise, the fixed-effects model was reported. For each outcome with data from five or more studies, we began the analysis by creating a funnel plot, comparing the magnitude of the relative risk on the horizontal axis with the standard error of the log relative risk on the vertical axis [20]. We used Begg's and Egger's tests [21] to detect possible publication biases. Data were considered significant if the probability of a chance occurrence was less than 5% (p = 0.05).

## Results

### Eligible RCTs

**Figure 1:**
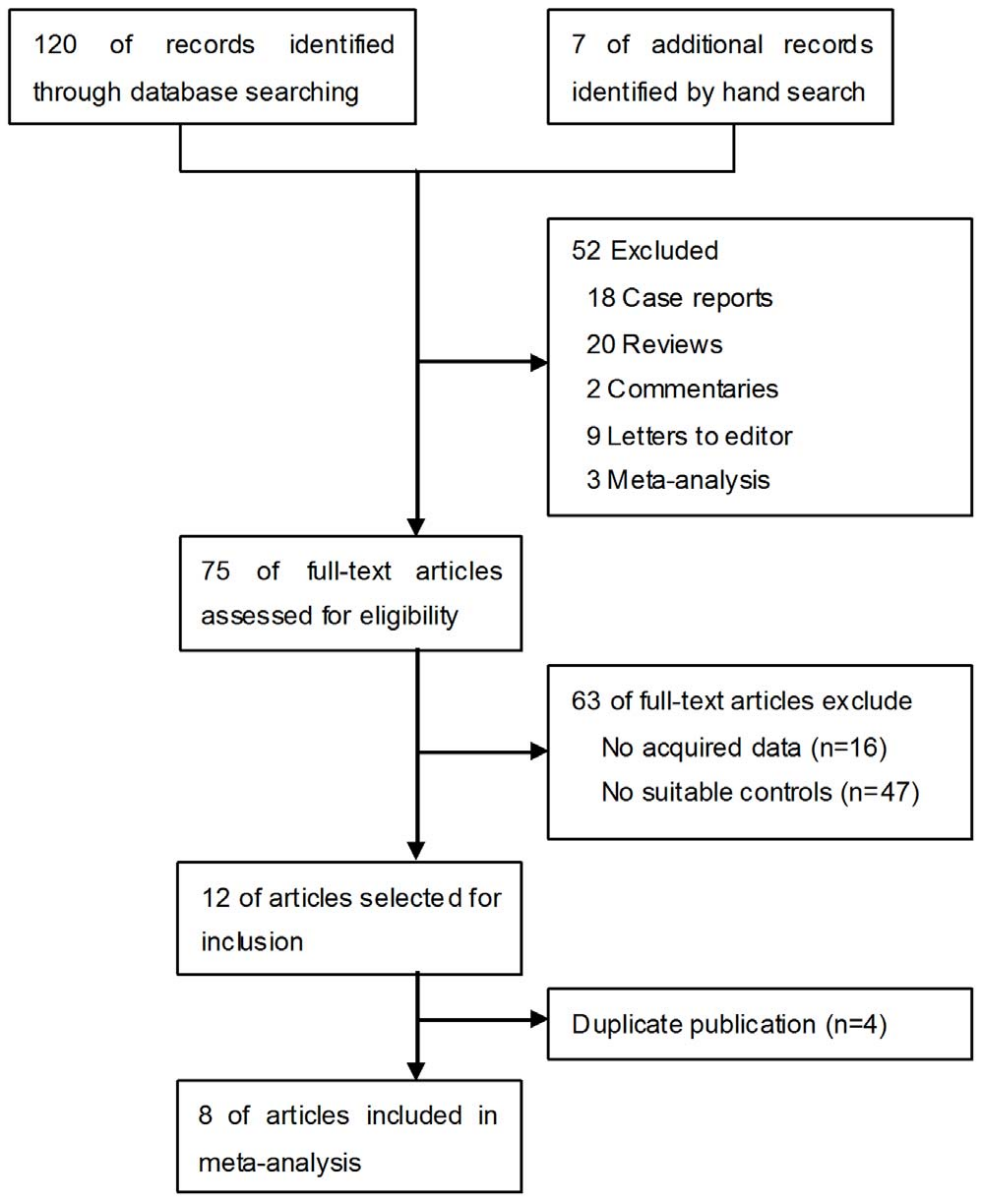
A flow chart showing the progress of trials through the review.

Our initial search strategy yielded a total of 120 potentially relevant clinical studies (see Figure 1 for the process of selecting these studies). Furthermore, 7 relevant papers were screened by hand from the reference lists. We excluded review articles, case reports, commentaries, letters to the editor and meta-analyses. After independent review, 75 publications that reported RCT results were considered to be eligible for inclusion in the analysis. Of these 75 publications, 16 were excluded because they did not provide data for calculating the odds ratio (OR), 47 were excluded because they did not include suitable control groups. Of the 12 studies remaining, 4 [22]–[25] were excluded because the same authors published several reports on the same patients or on select data from other published trials, and only the best-quality study was considered. Therefore, 8 [11], [14], [26]–[31] publications were available to analyze. This meta-analysis fully complied with the PRISMA Statement (see Checklist S1) for systematic reviews and meta-analyses.

### Study characteristics and quality

The baseline characteristics of the 8 RCTs included in the meta-analysis are listed in Table 1. These RCTs were all published after 1998. Five of the studies were published in English [11], [14], [26]–[28] and 3 of the studies were published in German [29]–[31]. A total of 622 patients from 8 randomized controlled trials were identified and included in the analysis.

**Table 1:** Study characteristics of the included RCTs studies.

Reference	Language	Sample Sizes		Patients Selection		Outcome	Critical Appraisal
		PPPD	PRPD	Inclusion	Exclusion		
**Kawai 2011**	English	64	66	♦Pancreatic head/periampullary carcinoma	♦Infiltration of the peripyloric LN or stomach	♦Survival	♦No description of sample size calculation
				♦Undergoing PD	♦Concomitant severe comorbidity	♦Complications	♦Proper reporting of sequential randomization
					♦Combined liver resection	♦DGE	♦Adequate definition of outcome parameters
					♦Proved mental illness	♦Weight	♦No description of allocation concealment/blinding
					♦Without an informed consent	♦QoL	♦In-house sequential follow-up
						♦Nutritional Status	♦No loss to follow up
							♦Short follow-up period
**Srinarmwong 2008**	English	14	13	♦Suspected pancreatic/ periampullary carcinoma	♦Previous gastric resection	♦Survival	♦No description of sample size calculation
					♦Distant metastases or locally unresectable tumors	♦Complications	♦Proper description of randomization process
					♦Pylorus/stomach involved	♦DGE	♦ Proper definition of outcome parameters
					♦ Refused to participate in the study	♦ Pathological findings	♦ No description of allocation concealment/blinding
							♦ No loss to follow up
							♦ Short and long follow-up period
**Lin 2005**	English	14	19	♦ Pancreatic head/periampullary carcinoma	—	♦ Survival	♦ No description of sample size calculation
				♦ Undergoing PD		♦ Complications	♦ Inadequate description of randomization process
						♦ DGE	♦ Insufficient definition of outcome parameters
							♦ No description of allocation concealment/blinding
							♦Follow-up not specified
							♦Short- and long-term results inconsistent
**Siler 2005**	English	64	66	♦Suspected pancreatic/ periampullary carcinoma	♦Previous gastric resection	♦Survival	♦Description of sample size calculation
				♦Considered resectable		♦Complications	♦Proper reporting of sequential randomization
				♦Suitable for surgery		♦DGE	♦Adequate definition of outcome parameters
						♦Weigt	♦Reporting in accordance with CONSORT
						♦QoL	♦Balanced study population
							♦Sequential, personal follow-up
							♦Allocation concealment unclear
							♦No specification of loss to follow-up
**Tran 2004**	English	87	83	♦Suspected pancreatic/ periampullary carcinoma	♦Previous gastric resection	♦Survival	♦Description of sample size calculation
				♦Considered resectable	♦Distant metastases	♦Complications	♦Adequate reporting of sequential randomization and allocation concealment
					♦Pylorus/stomach involved	♦DGE	♦Adequate definition of outcome parameters
					♦Positive peripyloric LN	♦Weight	♦Balanced study population
							♦Multicenter approach
							♦In-house sequential follow-up
							♦No loss to follow up
							♦ITT analysis
							♦Short follow-up period
**Wenger 1999**	German	24	24	♦Suspected pancreatic/periampullary carcinoma	♦Infiltration of the pylorus or stomach	♦Survival	♦No description of sample size calculation
				♦Considered resectable	♦Peritoneal carcinomatosis	♦Complications	♦Inadequate reporting on randomization process
					♦Age >75 y	♦DGE	♦Insufficient definition of outcome parameters
					♦Poor general health	♦Weight	♦Sequential, personal follow-up
						♦QoL	♦No description of allocation concealment/blinding
							♦Only R0 resections included/randomized (selection bias)
							♦Loss to follow-up 70.8% (PPW) and 58.3% (CW)
**Bloechle 1998**	German	23	21	♦Suspected periampullary carcinoma	—	♦Complications	♦No description of sample size calculation
				♦Stages cT1–4, cN0–1, cM0		♦DGE	♦No description of randomization process
						♦QoL	♦Insufficient definition of outcome parameters
							♦No description of allocation concealment/blinding
							♦No specification of follow-up
**Paquet 1998**	German	17	23	♦Suspected pancreatic/periampullary carcinoma	♦Contraindication to surgery	♦Survival	♦No description of sample size calculation
				♦Considered resectable	♦Metastases or advanced disease	♦Complications	♦Insufficient definition of outcome parameters
						♦DGE	♦Randomization process described
						♦Weight	♦Sequential, personal, long follow-up period
							♦No description of allocation concealment/blinding

Abbreviations and notes: —, not mentioned; DGE, delayed gastric empty; LN, lymph nodes; PD, pancreaticoduodenectomy; PPPD, pylorus-preserving pancreaticoduodenectomy; PRPD, pylorus-resection pancreaticoduodenectomy; QoL, quality of life; ITT, intention-to-treat analysis.

We extracted data for 13 postoperative outcomes from the studies. The results showed that possible publication bias may exist in DGE based on Begg's or Egger's test (Begg's test, p = 0.039; Egger's test, p = 0.003). And there are some data could not available for test such as biliary leakage and gastroenterostomy leakage (see Table 2). Ten outcomes had data from enough trials to create funnel plots (see Funnel plot S1 to S3); with the exception of DGE, all of the outcomes were symmetric.

**Table 2:** Egger's test and Begg's test of outcomes included in the meta-analysis.

	Mortality	Overall morbidity	Pancreatic fistula	Wound infection	Postoperative bleeding	Delayed gastric empty	Biliary leakage	Ascites	Gastroenterostomy leakage
Egger's test	0.787	0.280	0.425	0.575	0.523	0.003	NA	0.716	NA
Begg's test	0.497	1.0	0.573	0.624	1.0	0.039	NA	0.602	NA

Abbreviations and notes: NA, data not available.

### Long-term survival

**Figure 2:**
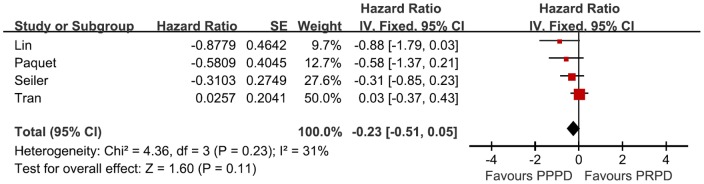
Forest plot of long-term survival. The 95% confidence interval (CI) for the hazard ratio for each study is represented by a horizontal line and the point estimate is represented by a square. The size of the square corresponds to the weight of the study in the meta-analysis. Pooled treatment effect is shown as a diamond that spans the 95% CI. Data for a fix-effects model are shown as there was low statistical heterogeneity (I^2^ = 31%). df  =  degrees of freedom; IV  =  Inverse Variance; I^2^ =  percentage of the total variation across studies due to heterogeneity; Z =  test of overall treatment effect.

Four RCTs were included for Long-term survival analysis [11], [27], [28], [31]. The meta-analysis revealed no difference in long-term survival between the PPPD and PRPD groups (Hazard Ratio [HR] = 0.23, p = 0.11) (Figure 2). One RCT [14] only described one- and two-year survival rates, and the results were also comparable for the two surgical procedures.

### Mortality and Morbidity

To identify the particular differences between PPPD and PRPD and exclude the influence of confounding factors, we calculated the overall relative risk for the mortality and morbidity from these RCTs, and the two groups were directly compared.

**Figure 3:**
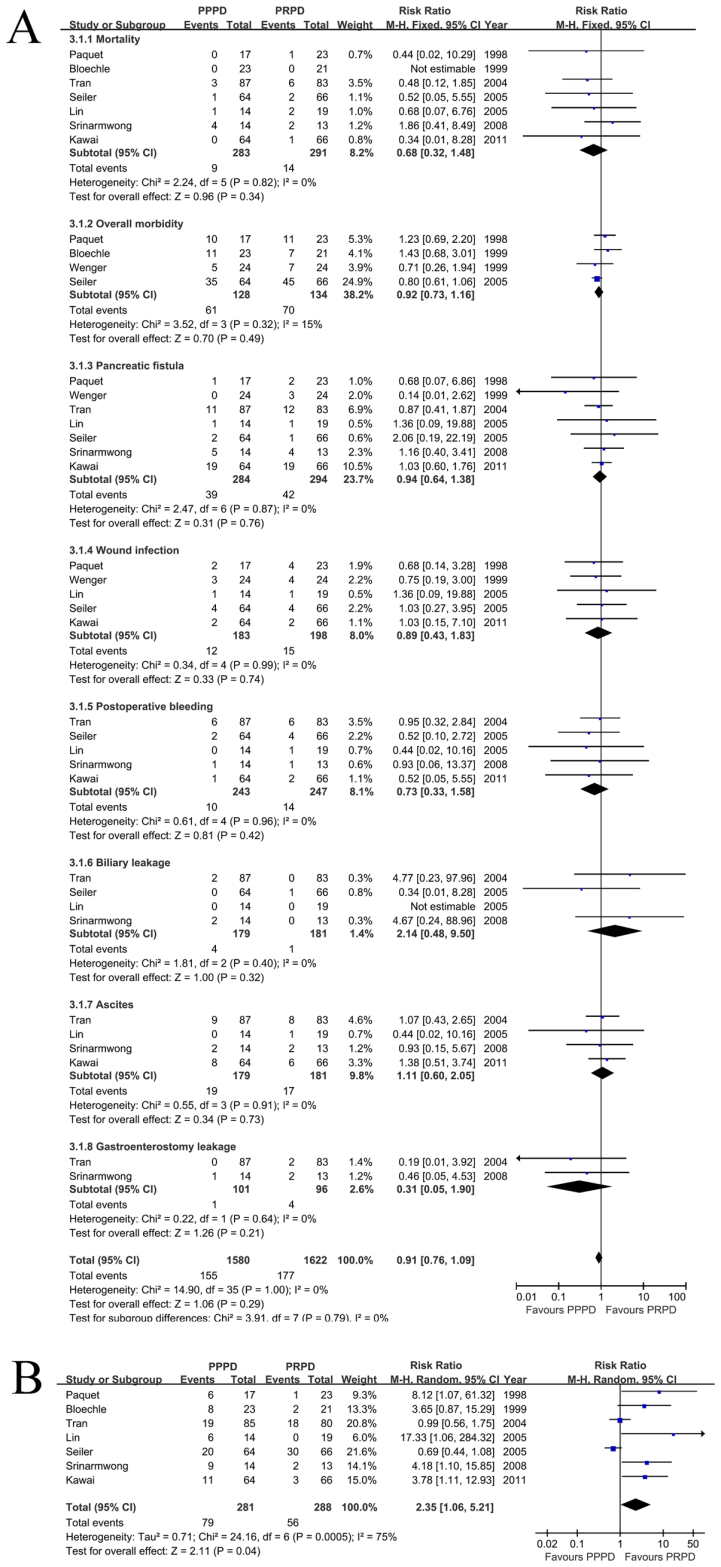
Forest plot of mortality and morbidity. The 95% confidence interval (CI) for the risk difference, risk ratio for each study is represented by a horizontal line and the point estimate is represented by a square. The size of the square corresponds to the weight of the study in the meta-analysis. The 95% CI for pooled estimates is represented by a diamond. df  =  degrees of freedom; MH  =  Mantel-Haenszel test; I^2^ =  percentage of the total variation across studies due to heterogeneity; Z =  test of overall treatment effect. A: mortality and morbidity for a fixed-effects model are shown as there were low statistical heterogeneity (I^2^<25%) (morbidity: including overall morbidity, pancreatic fistula, wound infection, postoperative bleeding, biliary leakage, ascites and gastroenterostomy leakage). B: Delayed gastric empty (DGE) for a random-effects model is shown as there was high statistical heterogeneity (I^2^ = 75%).

All the trials reported the mortality for a total of 574 patients. We performed a meta-analysis to calculate the overall RR associated with PPPD and PRPD. No heterogeneity was identified among the studies about mortality (Figure 3A, p = 0.82, I^2^ = 0%). Using a fixed-effects model, the overall RR for PPPD versus PRPD was 0.68 (95% CI, 0.32–1.48). Therefore, there was no significant difference in mortality.

The risks of overall morbidity, fistula, wound infection, postoperative bleeding, biliary leakage, ascites, and gastroenterostomy leakage did not differ significantly between the groups, although the confidence intervals for these outcomes were relatively wide (Figure 3A). Because the event rates were low, the confidence intervals were not wide in absolute terms. The results revealed a trend toward lower DGE rate (Figure 3B, RR = 2.35, p = 0.04, 95% CI, 1.06–5.21) with PRPD. There was substantial heterogeneity present in the outcomes (I^2^ = 75%, p = 0.0005). We were unable to account for the heterogeneity with any of the a priori hypotheses. The interaction test demonstrated no difference between these subgroups (I^2^ = 0%; p = 0.79).

### Operation related events

**Figure 4:**
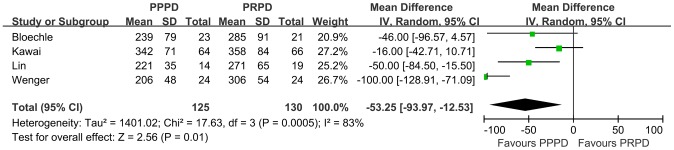
Forest plot of operating time. The 95% confidence interval (CI) for the hazard ratio for each study is represented by a horizontal line and the point estimate is represented by a square. The size of the square corresponds to the weight of the study in the meta-analysis. The 95% CI for pooled estimates is represented by a diamond. Data for a random-effects model is shown as there was high statistical heterogeneity (I^2^ = 83%). df  =  degrees of freedom; I^2^ =  percentage of the total variation across studies due to heterogeneity; IV =  Inverse Variance; Z =  test of overall treatment effect.

**Figure 5:**
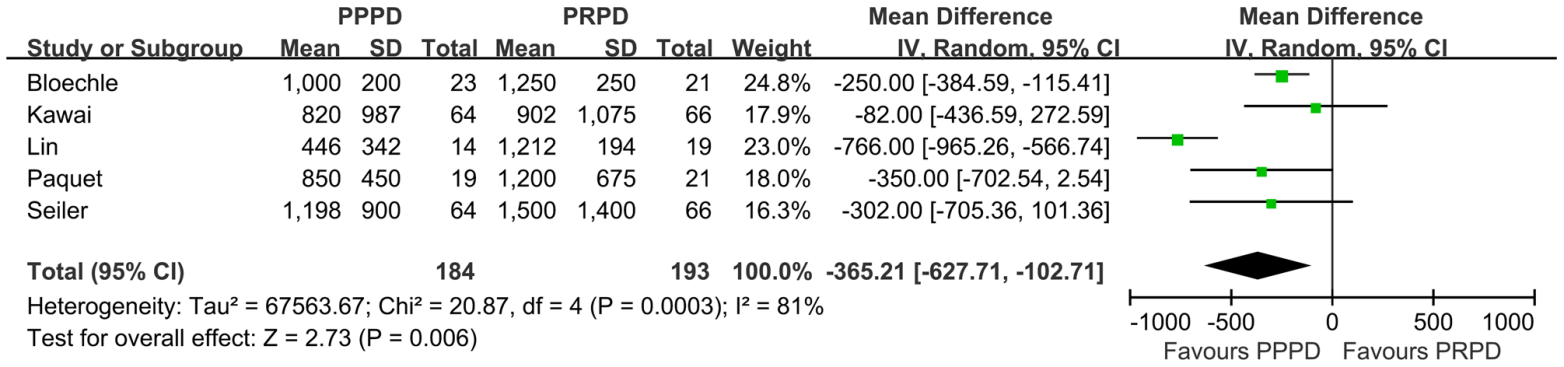
Forest plot of intraoperative blood loss. The 95% confidence interval (CI) for the hazard ratio for each study is represented by a horizontal line and the point estimate is represented by a square. The size of the square corresponds to the weight of the study in the meta-analysis. The 95% CI for pooled estimates is represented by a diamond. Data for a random-effects model is shown as there was high statistical heterogeneity (I^2^ = 81%). df  =  degrees of freedom; I^2^ =  percentage of the total variation across studies due to heterogeneity; IV =  Inverse Variance; Z =  test of overall treatment effect.

**Figure 6:**
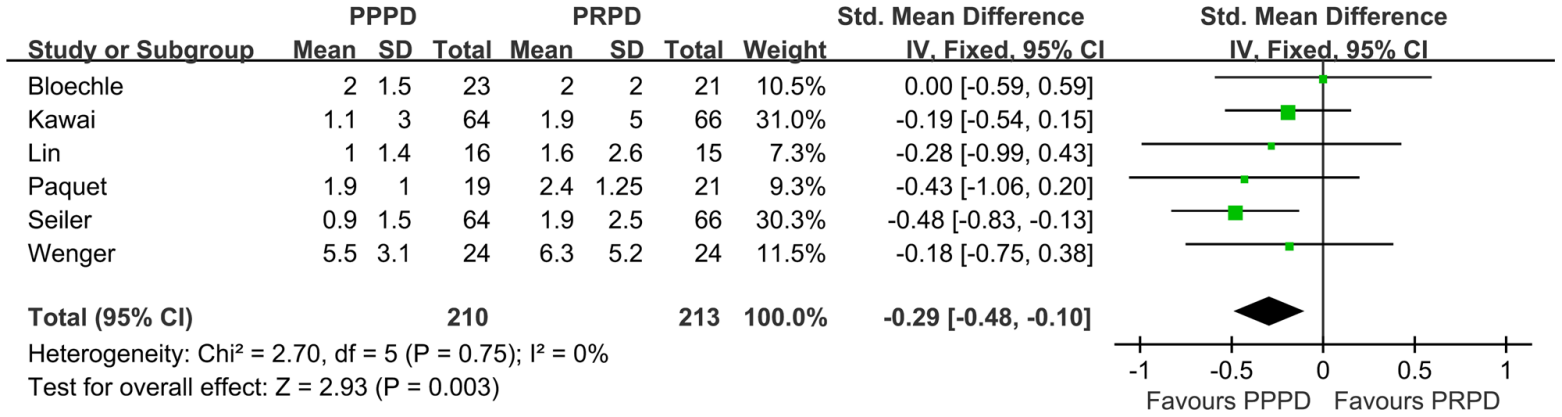
Forest plot of red blood cell transfusion. The 95% confidence interval (CI) for the hazard ratio for each study is represented by a horizontal line and the point estimate is represented by a square. The size of the square corresponds to the weight of the study in the meta-analysis. The 95% CI for pooled estimates is represented by a diamond. Data for a fix-effects model is shown as there was no statistical heterogeneity (I^2^ = 0%). df  =  degrees of freedom; I^2^ =  percentage of the total variation across studies due to heterogeneity; IV =  Inverse Variance; Z =  test of overall treatment effect.

**Figure 7:**
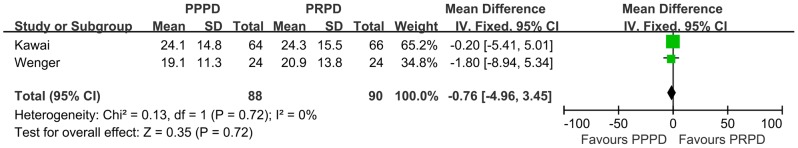
Forest plot of hospital stay. The 95% confidence interval (CI) for the hazard ratio for each study is represented by a horizontal line and the point estimate is represented by a square. The size of the square corresponds to the weight of the study in the meta-analysis. The 95% CI for pooled estimates is represented by a diamond. Data for a fix-effects model is shown as there was no statistical heterogeneity (I^2^ = 0%). df  =  degrees of freedom; I^2^ =  percentage of the total variation across studies due to heterogeneity; IV =  Inverse Variance; Z =  test of overall treatment effect.

We calculated the WMD for the Operation related events from these RCTs. In the 4 studies that reported the operating time, PPPDs were performed more quickly than PRPDs, with a WMD of 53.25 minutes (Figure 4, p = 0.01, 95% CI, 12.53–93.97). Similarly, there was less estimated intraoperative blood loss (Figure 5, WMD = 365.21 ml, p = 0.006, 95% CI, 102.71–627.71) and less red blood cell transfusions (Figure 6, WMD = 0.29 U, p = 0.003, 95% CI, 0.10–0.48) in patients undergoing PPPD. There was a nonsignificant trend toward a shorter hospital stay in the PPPD group, with a WMD of 0.76 day (Figure 7, p = 0.72, 95% CI, 3.45–4.96).

## Discussion

Pancreatic cancer is one of the most fatal malignant tumor with a particularly poor outcome [1], [2]. Two operation techniques are predominantly performed, PRPD and PPPD. PPPD is similar to PRPD except that the pylorus is preserved. At present, controversy remains as to whether PRPD or PPPD is the better choice for periampullary and pancreatic carcinoma.

Because PPPD involves a less extensive dissection, certain surgeons have expressed concern that it may result in a higher rate of tumor recurrence for pylorus-preserving procedure does not follow the rules of radical tumor surgery [6]. Roder *et al* reported a reduced survival rate after pylorus-preserving surgery in patients with pancreatic tumors and periampullary carcinoma respectively [10]. However, there is increasing evidence that the long-term survival is comparable for the pylorus-preserving and pylorus-resecting procedures [11], [27], [28], [32]. Several retrospective studies have revealed that the long-term survival is not influenced by the type of resection [33], [34]. Interestingly, a marginal benefit of PPPD for the treatment of pancreatic carcinoma has been observed in terms of long-term survival (p = 0.09) [5].

Resection margins and lymph node status were the most powerful prognostic factors for survival [35]. The risk of tumor spread in the duodenal resection margin is rare. Berthold *et al* reported that only 1 patient out of 50 total PD patients presented with a tumor nest in the proximal duodenum [36]. Clinical observations and pathological examination have indicated that the pancreatic resection and bile duct margins, rather than the duodenal margin, are more likely to be infiltrated by tumor [37]. The primary margin difference between PRPD and PPPD is that of the duodenal resection; the pancreatic and bile duct margin are similar. Therefore, it can be hypothesized that there is no difference in surgical margins between these two procedures. On the other hand, it was indicated that peripyloric and stomach lymph node metastasis is a rare condition in carcinoma of the pancreatic head [36]. These results were supported by study of Kawai *et al* [26] that demonstrated that none of the 139 eligible patients undergoing PD had peripyloric nodes metastasis.

In our meta-analysis, the long-term survival was not significantly different between the two techniques. However, there remained the potential for lymph node metastasis and duodenal involvement, and certain authors have reported that the frequency of peripyloric lymph node metastasis in pancreatic head carcinoma was up to 12% [38]. Therefore, PPPD could be acceptable for patients with pancreatic or periampullary carcinoma, and an intraoperative frozen section of the peripyloric lymph nodes or the duodenal resection margin would be necessary if tumor involvement was suspected.

Delayed gastric empty (DGE) is one of the most common complications after PD and has been reported to occur in 1∼6% of patients [3]. Although postoperative DGE is not life threatenting, it results in decreased quality of life, impaired of oral intake, increased hospital costs and the delayed initiation of adjuvant chemotherapy [39]. It has been thought that higher rates of DGE occur after PPPD than after a PRPD. However, the reported incidence of DGE after a PPPD or a PRPD remains controversial. Certain studies have indicated PPPD was associated with a higher DGE rate than was PRPD [26]. Critics have reported that the incidence of DGE was comparable for both surgical procedure [34], [40]. The pathogenesis of DGE after PPPD has not been fully ascertained, several factors have been related to the occurrence of DGE, including ischemia of the pylorus and duodenum, denervation of the pylorus ring, gastric dysrhythmias, and the absence of gastrointestinal hormone [41].

Two recently published meta-analysis indicated that a marginal benefit existed in terms of DGE rate for PPPD compared with PRPD for pancreatic and periampullary carcinoma [13], [42]. However, our results, which included two new RCTs, demonstrated that PPPD had a significantly higher DGE rate compared with PRPD. One reason for this trend was that both the newly included RCTs [14], [26] reported a higher DGE rate in the PPPD group, the pooled data indicated a significant difference in the DGE rate. On the other hand, one RCTs [26] described PPPD as a procedure preserving the entire stomach. However, the other seven RCTs [11], [14], [27]–[31] described PRPD as standard Whipple's PD (SWPD), which involves a partial resection of distal stomach. Whether heterogeneity of surgical procedure has a significant impact on the DGE rate is unclear.

Postoperative DGE can be treated conservatively. Low-doses erythromycin is commonly administered to reduce the incidence of DGE after PPPD [43]. Preservation of the right gastric artery and vagus nerve during surgery has been associated with a decreased rate of DGE [44]. Furthermore, antecolic duodenojejunostomy was reported to be a useful reconstruction method after PPPD to reduce the incidence of DGE [45]. Therefore, surgeons should undertake certain measures during the intra- and post-operative periods to decrease the rate of DGE.

The present meta-analysis indicated that there was no difference in mortality and other morbidity between both the two procedures. However, the PPPD procedure provided a significant benefit in terms of intra-operative blood loss, blood transfusions and operating time. The significant reduction in intra-operative blood loss and blood transfusions with PPPD created an advantage over PRPD in terms of cost, but the benefits regarding postoperative morbidity were unclear. Certain authors indicated that units of blood transfused appear to be an important factor for survival [46], but in our meta-analysis, long-term survival was not significantly different between the two procedures. Furthermore, although PPPD involved a less extensive dissection, some articles indicated that there were no significant differences in postoperative mortality between PPPD and PRPD [40], [47]. Other analysis did not identify any significant differences in the incidence of overall morbidity, pancreatic fistulas, wound infections, postoperative bleeding, biliary leakage, ascites and gastroenterostomy leakage between patients who underwent PPPD and those receiving the PRPD procedure, although the confidence intervals for these outcomes were relatively wide. Also there was no significant difference in hospital stay for PPPD versus PRPD.

There were particular limitations to our study. First, possible publication bias may exist in DGE based on Begg's and Egger's test. Three hypotheses explain this result: one, the heterogeneous definition of DGE may have created bias in the reported incidence rates; two, a possible contribution from individual operating technique differences; and three, the incidence of DGE was significantly heterogeneous in the included studies. We used the random-effects model to estimate the incidence may have minimized this bias. However, the results should be interpreted cautiously because of the above heterogeneity. Second, because the heterogeneous definition and the patients included in this analysis was limited, the confidence interval for morbidity such as ascites and anastomotic leakage were relatively wide. Third, we did not investigate the influence of adjuvant treatment such as chemotherapy or radiotherapy on the survival, mortality and morbidity for non available data. It is may another bias of the conclusion. Forth, another bias is the heterogenicity of surgical procedure. In the included articles, Kawai *et al* [26] reported PRPD with distal stomach preserved, whereas the other seven articles described PRPD as classical Whipple's PD, with distal stomach resection. This difference in PRPD procedure may cause bias of the postoperative outcome. Future studies should focus on the differences in the PRPD subgroups (with or without distal stomach resection) and then compare them with PPPD according to a randomized controlled trial protocol. Fifth, the present study has the typical limitations of meta-analytical methodology. Our findings and interpretations were limited by the quality and quantity of available data. An analysis of individual patient data would be more powerful to confirm our findings. Another concern is the possible existence of unpublished studies, which could lead to a potential publication bias, although we found no indication of such a bias using statistical methods designed to detect this particular type of bias. Finally, this was a meta-analysis at the study level, and confounding factors at the patient level could not be properly assessed and incorporated into the analysis.

## Conclusions

There were no significant differences in long-term survival, mortality, hospital stay or postoperative morbidity between the PPPD and PRPD groups for periampullary and pancreatic carcinoma. PPPD had certain advantages in terms of operating time, intraoperative blood loss and red blood cell transfusions, but presented with a significantly higher rate of DGE. Both surgical procedures are acceptable for the treatment of periampullary and pancreatic carcinoma. However, the results should be interpreted cautiously because the quality of the included studies was suboptimal.

## Supporting Information

Checklist S1 **Contains items including the title, abstract, methods, results, discussion and funding.**
(DOC)

Funnel plot S1 **Funnel plot of mortality, overall morbidity, wound infection, ascites, postoperative bleeding, gastroenterostomy leakage, pancreatic fistula and biliary leakage of patients undergoing PPPD and PRPD.**
(TIF)

Funnel plot S2 **Funnel plot of red blood cell transfusion of patients undergoing PPPD and PRPD.**
(TIF)

Funnel plot S3 **Funnel plot of intraoperative blood loss of patients undergoing PPPD and PRPD.**
(TIF)
