# Supplementary figures and images for: Pylorus-Preserving Versus Pylorus-Resecting Pancreaticoduodenectomy for Periampullary and Pancreatic Carcinoma: A Meta-Analysis
Source: PLoS One. 2014 Mar 6;9(3):e90316. doi: 10.1371/journal.pone.0090316 (PMC3946060; doi:10.1371/journal.pone.0090316)

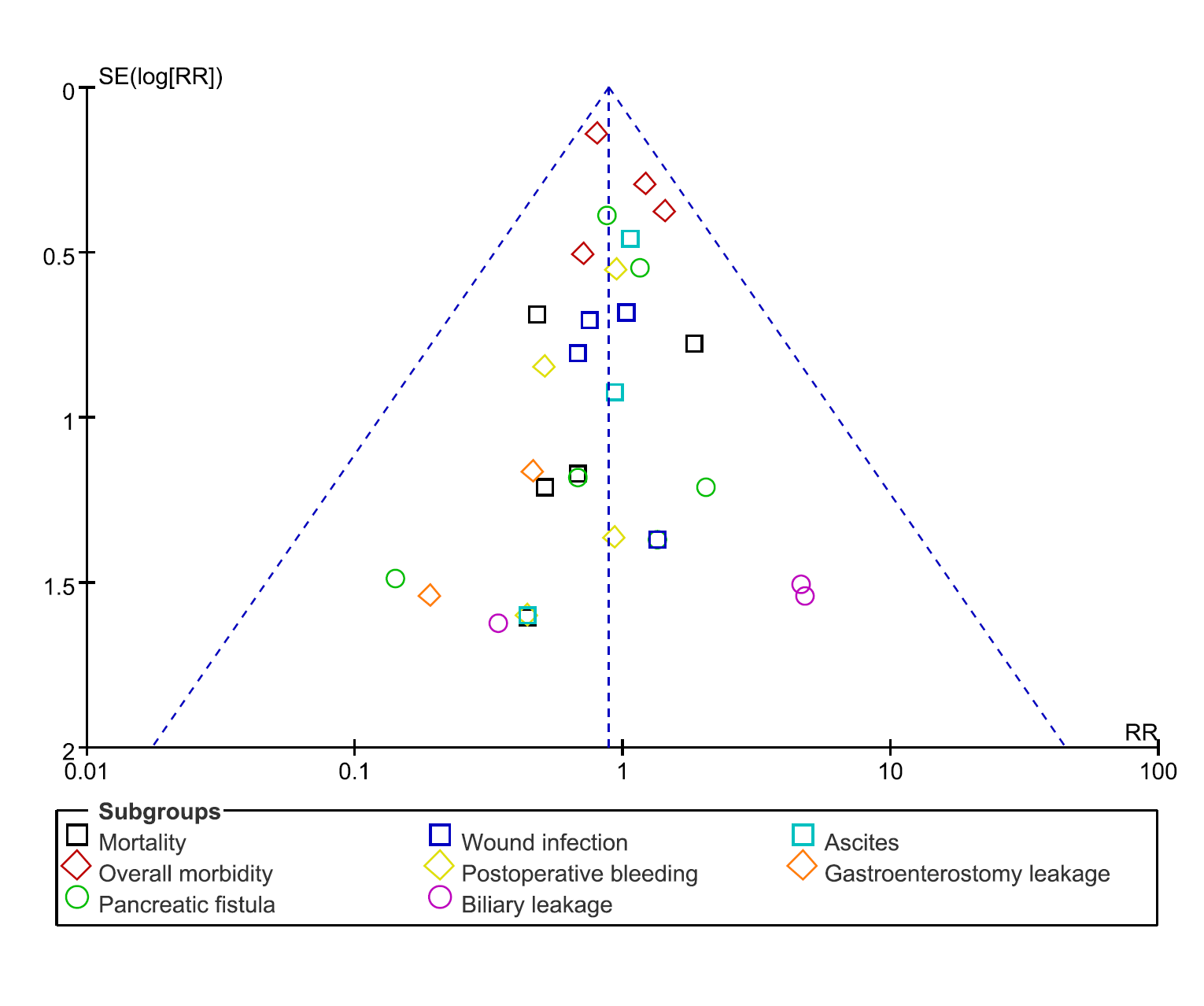

Supplement: Funnel plot S1 — Funnel plot of mortality, overall morbidity, wound infection, ascites, postoperative bleeding, gastroenterostomy leakage, pancreatic fistula and biliary leakage of patients undergoing PPPD and PRPD. (TIF) [file pone.0090316.s002.tif]

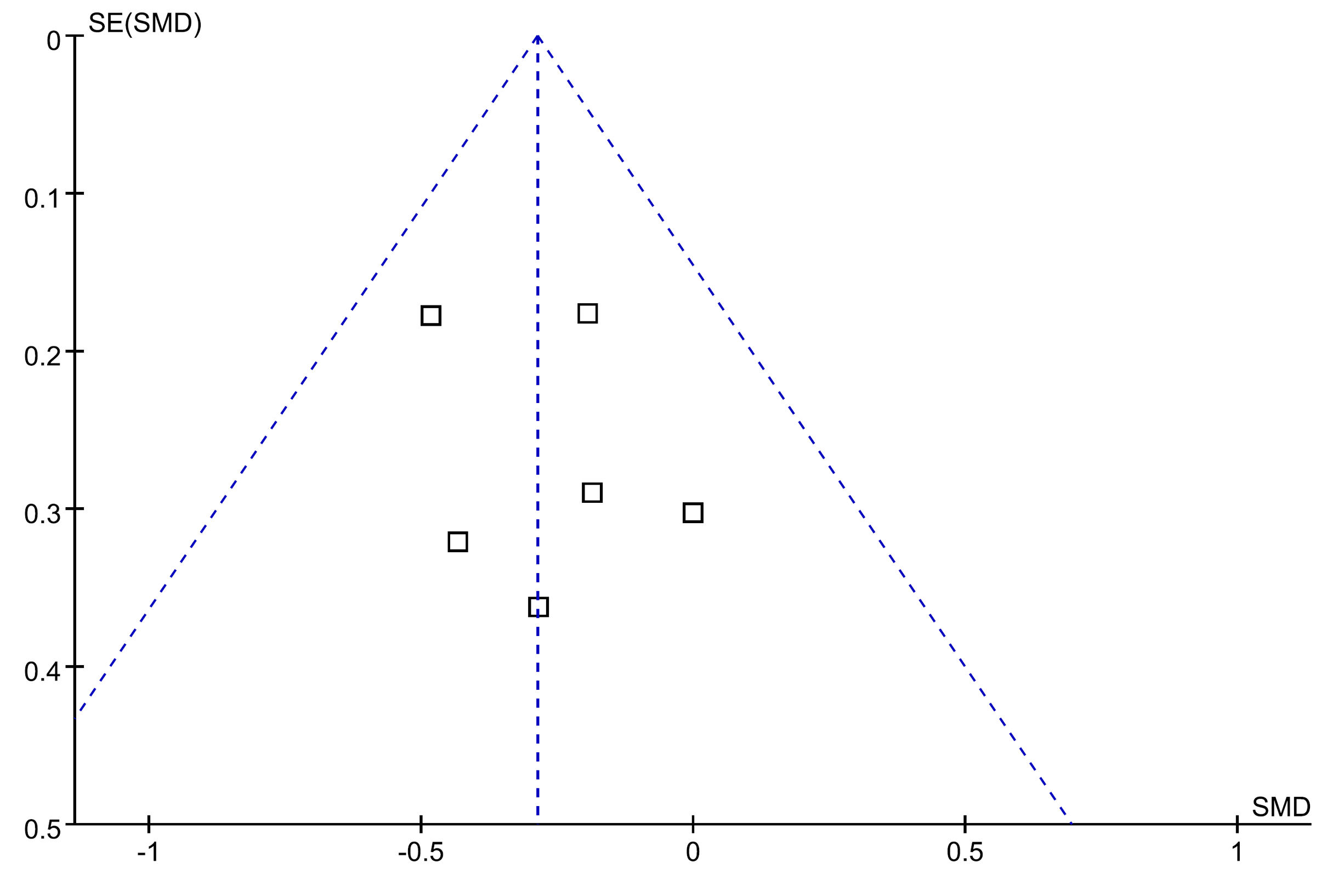

Supplement: Funnel plot S2 — Funnel plot of red blood cell transfusion of patients undergoing PPPD and PRPD. (TIF) [file pone.0090316.s003.tif]

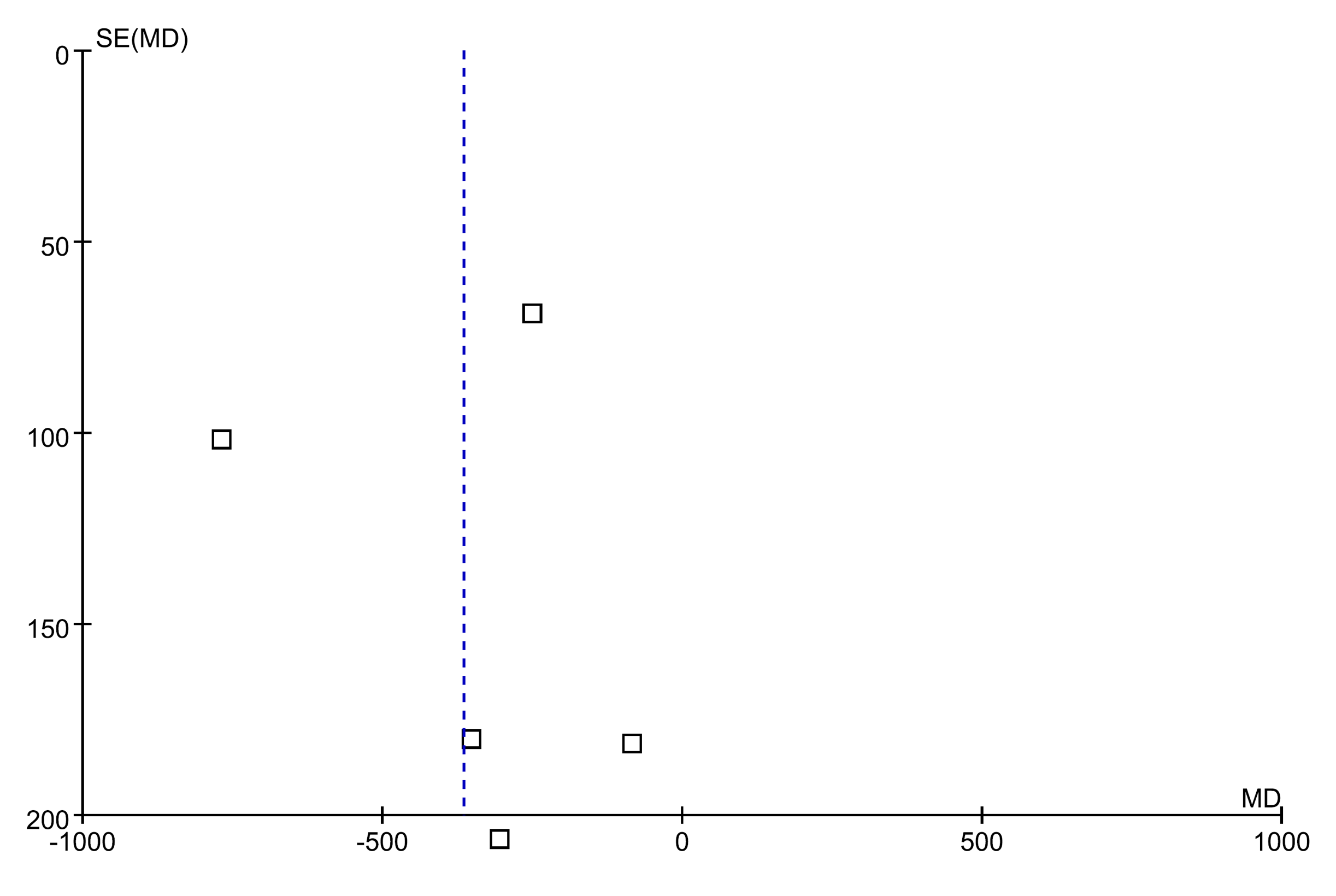

Supplement: Funnel plot S3 — Funnel plot of intraoperative blood loss of patients undergoing PPPD and PRPD. (TIF) [file pone.0090316.s004.tif]
